# Naïve Bayes Classifier with Feature Selection to Identify Phage Virion Proteins

**DOI:** 10.1155/2013/530696

**Published:** 2013-05-15

**Authors:** Peng-Mian Feng, Hui Ding, Wei Chen, Hao Lin

**Affiliations:** ^1^School of Public Health, Hebei United University, Tangshan 063000, China; ^2^Key Laboratory for Neuroinformation of Ministry of Education, Center of Bioinformatics, School of Life Science and Technology, University of Electronic Science and Technology of China, Chengdu 610054, China; ^3^Department of Physics, School of Sciences, Center for Genomics and Computational Biology, Hebei United University, Tangshan 063000, China

## Abstract

Knowledge about the protein composition of phage virions is a key step to understand the functions of phage virion proteins. However, the experimental method to identify virion proteins is time consuming and expensive. Thus, it is highly desirable to develop novel computational methods for phage virion protein identification. In this study, a Naïve Bayes based method was proposed to predict phage virion proteins using amino acid composition and dipeptide composition. In order to remove redundant information, a novel feature selection technique was employed to single out optimized features. In the jackknife test, the proposed method achieved an accuracy of 79.15% for phage virion and nonvirion proteins classification, which are superior to that of other state-of-the-art classifiers. These results indicate that the proposed method could be as an effective and promising high-throughput method in phage proteomics research.

## 1. Introduction

Phage is a virus that infects and replicates within bacteria. Phages are widely distributed in locations populated by bacterial hosts, such as soil or the intestines of animals. A complete infectious phage viral particle (also, namely, phage virion) consists of an inner core of nucleic acid which gives the virus infectivity and a protein coat (called a capsid) which encases the nucleic acid and provides specificity, that is, determines which organisms the virus can infect. 

The nucleic acid of phage virions is either RNA or DNA. Proteins of phage virions include structural proteins and nonstructural proteins. Structural proteins commonly termed “phage virion proteins” are essential materials of the infectious viral particles, including shell proteins, envelope proteins, and virus particle enzymes. Nonstructural proteins (namely, phage nonvirion proteins) refer to that encoded by the viral genome and play important roles in biological process of viral genome replication and expression, but they do not bind to phage virions. Due to the distinct functions between phage virion proteins and phage nonvirion proteins, knowledge about the protein composition of phage virions is an essential step to further understand the functions of phage virions.

Although the use of mass spectrometry (MS) for the identification of phage virion proteins has become popular [1], it has not kept pace with the explosive growth of protein sequences generated in the postgenomic age. Hence, it is highly desired to develop automated methods for timely and reliably classifying the protein composition of phage virions.

To the best of our knowledge, there is no computational system for the classification of phage virion proteins. In the current study, we propose a Naïve Bayes based computational model for predicting phage virion proteins using amino acid compositions and dipeptide compositions. The correlation-based feature subset selection algorithm [2] was introduced to find the optimal feature set. By using the optimized features, the proposed model was evaluated in a benchmark dataset in the jackknife test. The performance demonstrates that this model could be a potentially useful tool for the annotation of the phage proteins.

According to some recent comprehensive reviews [3, 4] and demonstrated by a series of recent publications [5–10], to establish a really useful statistical predictor, we need to consider the following procedures: (i) construct or select a valid benchmark dataset to train and test the predictor; (ii) formulate the statistical samples with an effective mathematical expression that can truly reflect their intrinsic correlation with the target to be predicted; (iii) introduce or develop a powerful algorithm (or engine) to operate the prediction; (iv) properly perform cross-validation tests to objectively evaluate the anticipated accuracy of the predictor; (v) establish a user-friendly web server for the predictor that is accessible to the public. In the following, let us describe how to deal with these steps one by one.

## 2. Materials and Methods

### 2.1. Dataset

The raw datasets adopted in this research were extracted from the UniProt [11]. For the purpose of obtaining a reliable benchmark dataset, the following steps were considered. Firstly, only the experimentally confirmed phage virion and phage nonvirion protein sequences were included. Secondly, the sequences which are fragments of other proteins were dislodged. Thirdly, sequences containing nonstandard letters, that is, “B,” “X,” or “Z,” were excluded as their meanings are ambiguous. After following the previous strict screening procedures, we obtained 121 phage virion protein sequences and 231 phage nonvirion protein sequences.

To prepare a high quality dataset, the CD-HIT program [12] was used to prune the data. By setting the cutoff of sequence identity to 40%, 307 sequences were remained in the final benchmark dataset, including 99 phage virion protein sequences and 208 phage nonvirion protein sequences.

### 2.2. Feature Vector

One of the most important parts for identifying protein attributes is to generate a set of proper informative parameters to encode the protein sequences. To avoid completely losing the sequence-order information, the pseudo amino acid composition (PseAAC) was proposed [13, 14] to replace the simple amino acid composition (AAC) for representing the sample of a protein. Since the concept of PseAAC was proposed in 2001 [13], it has been widely used to study various attributes of proteins, such as identifying bacterial virulent proteins [15], predicting supersecondary structure [16], predicting protein subcellular location [16–19], predicting membrane protein types [20], discriminating outer membrane proteins [21], identifying antibacterial peptides [22], identifying allergenic proteins [23], predicting metalloproteinase family [24], predicting protein structural class [25], identifying GPCRs and their types [26], identifying protein quaternary structural attributes [27], predicting protein submitochondria locations [28], identifying risk type of human papillomaviruses [29], identifying cyclin proteins [30], predicting GABA(A) receptor proteins [31], and classifying amino acids [32], among many others (see a long list of papers cited in the References section of [3]). Recently, the concept of PseAAC was further extended to represent the feature vectors of DNA and nucleotides [7, 9], as well as other biological samples (see, e.g., [33, 34]). Because it has been widely and increasingly used, recently two powerful softwares, called “PseAAC-Builder” [35] and “propy” [36], were established for generating various special Chou's pseudoamino acid compositions.

The amino acid composition and dipeptide composition are the general forms of PseAAC and the simplest parameters, which also have been widely applied in the realm of protein prediction [37–40]. Hence, every protein sequence in the benchmark dataset was encoded in a discrete vector as

(1) $$\begin{array}{c} F={\left[f_{1},f_{2},\ldots ,f_{420}\right]}^{T}, \end{array}$$

where *f*_*i*_ is the normalized occurrence frequencies of the 20 amino acids (*i* = 1, 2,…, 20) and the 400 dipeptides (*i* = 21, 22, …, 420) in the protein sequence, respectively. *T* is the transposing operator.

### 2.3. Feature Selection

Inclusion of redundant and noisy features in the model building process would cause poor predictive performance and increased computation. Feature selection is the process of removing irrelevant features and is extremely useful in reducing the dimensionality of the data and improving the predictive accuracy. To reduce the dimension of the feature space and improve the precision of phage virion and nonvirion protein classification, the filter method Correlation-based Feature Selection [2] combined with Best-first search strategy was used in the process of feature selection in the current work.

The process starts with an empty set of features and generates all possible single feature expansions. The subset with the highest accuracy is chosen and expanded in the same way by adding single features. If the accuracy does not maximize with the expansion of a subset, the search drops back to the next best unexpanded subset and continues from there until all features are added. The subset with the highest accuracy will be selected as the final optimized feature set [41].

### 2.4. Naïve Bayes

Naïve Bayes is an effective statistical classification algorithm [42] and has been successfully used in the realm of bioinformatics [43–46]. The basic theory of Naïve Bayes is similar to that of Covariance Determinant (CD) [47–52]. But for Naïve Bayes, it assumes the attribute variables to be independent from each other given the outcome. This assumption greatly simplifies the calculation of conditional probabilities and also overcomes the divergent problem when using the CD prediction engine to deal with those systems in which the components of constituent feature vectors are normalized.

In the Naïve Bayes framework, a classification problem can be seen as the problem of finding the outcome with maximum probability given a set of observed variables. Given a phage viral protein example, described by its feature vector **F** = (*f*_1_, *f*_2_,…, *f*_*n*_), we are looking for a class **C** that maximizes the likelihood **P**(**F** | **C**) = **P**(*f*_1_, *f*_2_,…, *f*_*n*_ | **C**). Since the current work is intend to classify phage virion and nonvirion proteins, a binary class **C** ∈ {0,1} was generated, where 1 denotes that the sample was predicted as a phage virion protein and 0 denotes phage nonvirion protein. For the binary classification, the class for the protein sample could be determined by comparing two posteriors as

(2) P(C=1 ∣ F=f1,f2,…,fn)P(C=0 ∣ F=f1,f2,…,fn) =P(C=1)∏i=1nPi(fi ∣ C=1)P(C=0)∏i=1nPi(fi ∣ C=0).

Taking the logarithm of (2), we obtain

(3) log⁡P(C=1 ∣ F=f1,f2,…,fn)P(C=0 ∣ F=f1,f2,…,fn) =log⁡P(C=1)P(C=0)+∑i=1nlog⁡Pi(fi ∣ C=1)Pi(fi ∣ C=0).

Hence the sample will be predicted as 1 (phage virion protein) if

(4) $$\begin{array}{c} \log\frac{P\left(C=1\;|\;F=f_{1},f_{2},\ldots ,f_{n}\right)}{P\left(C=0\;|\;F=f_{1},f_{2},\ldots ,f_{n}\right)}\geq \theta \end{array}$$

and 0 (phage nonvirion protein) for otherwise. *θ* is the threshold determining the trade-off between sensitivity and specificity and can be trained on the training dataset to maximize the prediction performance.

### 2.5. Performance Evaluation

The performance of the proposed model was evaluated using sensitivity (Sn), specificity (Sp), and accuracy (Acc), which are expressed as

(5) $$\begin{array}{c} \text{Sn}=\frac{\text{TP}}{\text{TP}+\text{FN}},\\ \text{Sp}=\frac{\text{TN}}{\text{TN}+\text{FP}},\\ \text{Acc}=\frac{\text{TP}+\text{TN}}{\text{TP}+\text{FN}+\text{TN}+\text{FP}}. \end{array}$$

TP, TN, FP, and FN represent the number of the correctly recognized phage virion proteins, the number of the correctly recognized phage nonvirion proteins, the number of phage nonvirion proteins recognized as phage virion proteins, and the number of phage virion proteins recognized as phage nonvirion proteins, respectively. 

As the performance of the current classifier depends on the threshold *θ* as given in (4), the threshold independent parameter, receiver operating characteristic curve, was employed as well. Therefore, the quality of a classifier can be objectively evaluated by measuring the area under the receiver operating characteristic curve (auROC). The value of auROC score ranges from 0 to 1, with a score of 0.5 corresponding to a random guess and a score of 1.0 indicating a perfect separation.

## 3. Results and Discussion

Three cross-validation methods, namely, subsampling test, independent dataset test, and jackknife test, are often employed to evaluate the predictive capability of a predictor. Among the three methods, the jackknife test is deemed the most objective and rigorous one that can always yield a unique outcome as demonstrated by a penetrating analysis in a recent comprehensive review [53] and hence has been widely and increasingly adopted by investigators to examine the quality of various predictors (see, e.g., [7, 19, 21, 30, 54–56]). Accordingly, the jackknife test was used to examine the performance of the model proposed in the current study. In the jackknife test, each sequence in the training dataset is in turn singled out as an independent test sample, and all the rule parameters are calculated without including the one being identified.

### 3.1. Prediction of Phage Virion Proteins

We trained the Naïve Bayes classifier using Waikato Environment for Knowledge Analysis (WEKA) [57] on the benchmark dataset. As shown in Table 1, an auROC score of 0.758 and an accuracy of 75.57% with an average sensitivity of 53.54% and an average specificity of 83.17% were obtained for the classification of phage virion and nonvirion proteins by using all the 420 features, that is, 20 amino acid compositions and 400 dipeptide compositions.

In order to identify prominent features that can distinguish between phage virion and nonvirion proteins, feature selection method as introduced in Section 2.3 was carried out to eliminate the redundant features using WEKA in a tenfold cross-validation approach on the benchmark dataset. We found that the proposed method achieved a maximum accuracy of 79.48% and auROC of 0.86 when the feature dimension reduced to 38 (i.e., V, T, A, H, K, E, R, S, LE, VT, VG, MK, TA, TS, AT, HI, KL, KI, KH, KN, KK, KD, KE, KW, KR, DK, EF, EL, EV, EK, EE, EW, CE, WK, RE, SG, GV, and GG). The jackknife test results of the Naïve Bayes classifier based on the 38 optimized features were listed in Table 1. As it can be seen from Table 1, the current method yielded a best auROC score of 0.855 and a predictive accuracy of 79.15% with an average sensitivity of 75.76% and an average specificity of 80.77% (Table 1). Both predictive accuracy and auROC are higher than that of the model based on the 420 features.

### 3.2. Comparison with Other Methods

To the best of our knowledge, there exists no theoretical method for phage virion and nonvirion protein classifications. Therefore, we cannot provide the comparison analysis with published results to confirm that the model proposed here is superior to other methods. However, the proposed Naïve Bayes classifier was compared with other state-of-the-art classifiers, that is, BayesNet, RBFnetwork, Random Forest, J48, Support Vector Machine (SVM), and LogitBoot. All the classifiers were compared on the benchmark dataset based on the optimized features (i.e., V, T, A, H, K, E, R, S, LE, VT, VG, MK, TA, TS, AT, HI, KL, KI, KH, KN, KK, KD, KE, KW, KR, DK, EF, EL, EV, EK, EE, EW, CE, WK, RE, SG, GV, and GG). Their best predictive results from jackknife test were shown in Table 2.

The predictive accuracy of Naïve Bayes is approximately 3%, 4%, 5%, and 7% higher than that of the BayesNet, Random Forest, LogitBoot, and J48 classifiers, respectively. Although the accuracies of RBFnetwork and SVM are equal to that of Naïve Bayes, their auROC scores are lower than that of Naïve Bayes. These results indicate that the proposed Naïve Bayes model can be effectively used to classify phage virion and nonvirion proteins.

## 4. Conclusions

In this study, the Naïve Bayes classifier with feature selection method is presented to identity phage virion proteins based on the primary sequence information. By using Correlation-based Feature Subset Selection algorithm, the feature dimensions were reduced, and 38 prominent features that could remarkably improve the predictive accuracies were obtained. However, the detailed analyses of the selected features are required to provide more information about their roles in biological activity. The accuracy for the classification of phage virion and nonvirion proteins reached 79.15% in the jackknife test, indicating that the proposed method is an effective tool for phage virion protein identification. It is expected that the presented model will provide novel insights into the research on phage proteomics. Since user-friendly and publicly accessible web servers represent the future direction for developing practically more useful predictors [58], we shall make efforts in our future work to provide a web server for the method presented in this paper.

## Figures and Tables

**Table 1:** Predictive performance of Naïve Bayes based on different features.

Feature dimensions	Sn (%)	Sp (%)	Acc (%)	auROC
420	53.54	83.17	75.57	0.758
38	75.76	80.77	79.15	0.855

**Table 2:** Comparison of Naïve Bayes with other methods by using optimized features.

Classifier	Sn (%)	Sp (%)	Acc (%)	auROC
BayesNet	68.69	79.81	76.22	0.799
RBFnetwork	72.73	82.21	79.15	0.839
Random Forest	55.56	84.62	75.24	0.802
LogitBoot	52.53	85.10	74.59	0.795
SVM	63.64	86.54	79.15	0.836
J48	61.62	77.88	72.64	0.671
Naïve Bayes	75.76	80.77	79.15	0.855
